# Multicellular Mechanoreciprocity in the Heart: Coordinated ECM Sensing and Remodeling by Cardiomyocytes, Fibroblasts, and Macrophages

**DOI:** 10.3390/cells15090773

**Published:** 2026-04-25

**Authors:** Colleen M. Simmerly, Robert E. Akins, Elise A. Corbin

**Affiliations:** 1 Department of Biomedical Engineering, University of Delaware, Newark, DE 19711, USA; csimmerl@udel.edu; 2 Department of Biomedical Research, Nemours Children’s Hospital, Wilmington, DE 19803, USA; robert.akins@nemours.org

**Keywords:** mechanoreciprocity, mechanosensing, dynamic stiffness, mechanobiology, extracellular matrix, cardiac biology

## Abstract

**Highlights:**

**What are the main findings?**

- Mechanobiology frameworks predominantly treat cell–ECM interactions as unidirectional instead of reciprocal.
- Existing biomaterial-based research models mimic isolated mechanical cues but often fail to recapitulate dynamic, multicellular interactions.

**What are the implications of the main findings?**

- Incorporating multicellular mechanoreciprocity is essential for mechanobiology studies to increase the physiologic relevance of disease modeling.
- Mechanoreciprocity provides a powerful, underexplored avenue for understanding and studying pathological remodeling.

**Abstract:**

The cardiac extracellular matrix (ECM) is a dynamic, mechanically active network continuously shaped and interpreted by cardiomyocytes, fibroblasts, and macrophages. Interdependent mechanosensing, force transmission, and ECM remodeling functions create multicellular feedback loops that control tissue stiffness, alignment, maturation, and fibrotic remodeling. Together, these biomechanical processes create reciprocal signaling pathways in which cellular behavior modifies the ECM while the ECM’s mechanics concurrently shape cellular phenotype and function. This review explores cell–ECM mechanoreciprocity, a physiologic framework that unifies cell-sensing mechanotransduction, mechano-electrical coupling, and ECM-based biochemical signaling with cell-driven ECM remodeling. We propose three interconnected feedback loops that integrate biochemical and mechanical cues across cell types: load amplification, structural alignment, and immune regulation. We discuss how advanced two- and three-dimensional engineered cardiac systems incorporating tunable and dynamic mechanical cues can be used to model these interactions. We address the limitations of existing experimental platforms and the need for better models to fully recapitulate in vivo complexities. Understanding and recreating these reciprocal mechanical interactions will provide essential frameworks for disease modeling and therapeutic development while reducing reliance on in vivo studies.

## 1. From ECM Structure to Multicellular Mechanoreciprocity

In this review, we discuss a systems-level framework called “mechanoreciprocity,” defined as the dynamic, multicellular exchange of mechanical and biochemical information between multiple cardiac cell types and the cardiac extracellular matrix (ECM). Cell interactions with neighboring cells and their extracellular environment are fundamental to cardiac function across scales, from single cells to the whole organ. The cardiac ECM provides structural support while actively regulating cell behavior through mechanical and biochemical signals that are continuously sensed and interpreted by cells. Cardiomyocytes bind ECM components such as collagens, laminins, and fibronectin through specialized adhesion complexes, integrating contractile activity with the surrounding matrix [1,2,3,4]. The composition and organization of the cardiac ECM are dynamically remodeled during both physiologic and pathologic conditions, through changes in ECM composition, ligand availability, and mechanical properties that alter cell–matrix interactions, driving region- and context-specific responses [1,5,6,7,8,9,10,11,12,13,14,15,16,17].

Mechanically, the ECM provides the structural basis for the complex bidirectional transmission of forces throughout the myocardium. Foundational work by Thomas K. Borg established that the cardiac ECM functions as a mechanically integrated, anisotropic network that distributes forces across the myocardium and supports coordinated tissue function [1,13]. Importantly, collagen fiber alignment enables force transmission between neighboring cells and across myocardial layers, coupling cardiomyocyte contraction to tissue-level mechanics [1,18]. While these findings established how forces are transmitted through the matrix, they do not fully explain how these mechanical signals are integrated and spread across multiple cell types.

The phenomena of mechanotransduction, ECM remodeling, and inflammatory signaling have each been extensively studied, and recent work has provided detailed insight into cardiomyocyte mechanics, fibroblast-driven matrix remodeling, and immune regulation of cardiac injury. However, these processes are most often studied as isolated or pairwise interactions. Prior reviews have addressed individual components of the cardiac biomechanical system, especially ECM composition across development and injury [19], ECM macromolecule dynamics in heart failure [20], and cell–cell interactions among cardiomyocytes, fibroblasts, and immune cells [21], but the conceptual frameworks in these reviews were delimited by developmental stage, injury type, or signaling pathway rather than as part of a continuous, integrated, mechanoreciprocal feedback system. Current frameworks do not fully incorporate the coordinated mechanical and biochemical signals across critical cell types within a shared ECM that produce tissue-level outcomes such as fibrosis, anisotropic organization, and contractile dysfunction. Recent in vivo studies also demonstrate coordinated cardiomyocyte–macrophage–ECM interactions in specific contexts such as cardiac regeneration [22], but these studies focused on specific cellular mechanisms rather than on multiscale interactions.

Importantly, the ECM encodes prior cellular activity through changes in composition, topology, ligand presentation, and viscoelasticity, and in turn regulates future cellular responses through adhesion dynamics, cytoskeletal tension, transcriptional programs, controlling cell fate. These interactions are inherently multicellular, emerging from the coordinated activity of cardiomyocytes, fibroblasts, macrophages, and other stromal populations that collectively sense and remodel a shared matrix.

The mechanoreciprocity framework overcomes the limitations of prior models by considering cardiac function and remodeling as being controlled by three interdependent feedback loops: (i) load amplification, (ii) fiber alignment, and (iii) immune remodeling. The load amplification loop couples cardiomyocyte contraction to fibroblast-mediated CM stiffening; the alignment loop reinforces anisotropic organization through coordinated force transmission; and the immune remodeling loop integrates mechanosensitive macrophage signaling with fibroblast activation and cardiomyocyte function. These three dynamically coupled feedback loops drive either homeostatic or maladaptive signaling cascades [11,23,24,25]. Although mechanoreciprocity is a feature of many load-bearing tissues, its importance is amplified in the heart due to continuous cyclic loading, which intensifies the need for coordinated ECM sensing and remodeling [26,27,28,29,30]. In this review, we use the mechanoreciprocity perspective to integrate molecular, cellular, and tissue-level mechanisms and to highlight how engineered cardiac systems can be leveraged to interrogate multicellular mechanical feedback in health and disease.

## 2. Structural and Molecular Architecture of ECM-Cardiac Cell Signaling

### 2.1. ECM Composition and Anisotropic Organization

The composition and organization of the ECM define how mechanical signals are transmitted and interpreted across cell types, establishing the structural basis for multicellular mechanoreciprocity. The ECM is a 3D fibrous network consisting primarily of type I collagen, additional crosslinked collagens, and a basement membrane containing adhesion proteins such as fibronectin, collagen type IV, laminin, and proteoglycans [8,31]. These components collectively regulate tissue mechanics through their organization, composition, and crosslinking.

Fibrillar collagens, the primary structural proteins within the cardiac ECM, influence cell behavior through both the degree of crosslinking and fiber alignment. Cells sense and align with local structures (contact guidance) [23,32] and actively remodel the matrix by depositing and reorganizing fibers parallel to the orientation of their cytoskeleton, creating a self-reinforcing alignment feedback loop [23]. This bidirectional interaction underlies ECM anisotropy, which is necessary for efficient cardiac force transmission across scales [23,24,33,34,35,36].

In addition to collagen alignment, the extent of collagen crosslinking strongly influences cell behavior by altering the ECM elastic modulus. Throughout this review, we use “stiffness” as a general term for a material’s resistance to deformation, typically corresponding to an effective elastic modulus unless otherwise specified. Stiffness emerges from the combined effects of ECM composition, organization, and crosslinking, and varies across physiologic states [20,37,38]. The myocardium contains spatially heterogeneous mechanical environments that optimize regional function. Healthy adult ventricular myocardium exhibits elastic moduli in the range of 10–15 kPa; whereas, fibrotic or infarcted myocardium frequently exceeds 20–50 kPa [37,39]. These changes in stiffness directly regulate cellular behavior by altering force transmission and resistance to deformation.

Importantly, in addition to providing mechanical cues, the binding of specific ECM proteins acts as a signal to cells [40]. ECM-based biochemical signals can alter cell behavior; for example, ECM ligand presentation can dictate myofibroblast activation state, even overcoming matrix stiffness cues [40]. In context-specific ways, the interactions between cells and specific ECM components can alter cell spreading, contractility, and extracellular adhesions [41]. Overall, ECM composition and organization are dynamically shaped by cell-mediated remodeling, linking matrix structure with cellular behavior.

### 2.2. Focal Adhesions, Costameres, and Integrins

In mechanoreciprocity, cells must not only generate force but must also continuously read and respond to the mechanical state of the surrounding matrix. Focal adhesions, costameres, and integrins function as key mechanotransductive structures linking the ECM, cytoskeleton, and gene expression, enabling cells to interpret and respond to their mechanical environment.

Focal adhesions join the actin cytoskeleton to ECM proteins through multiprotein complexes, which include integrins, vinculin, paxillin, talin, and focal adhesion kinase, among other proteins [42]. These structures remodel in response to mechanical cues, with stiffer environments promoting larger and more stable adhesions that enhance force transmission and cytoskeletal organization [42,43,44,45,46,47]. Focal adhesions are active participants in the load amplification loop: As fibroblast-driven ECM stiffening increases, adhesion reinforcement amplifies intracellular tension in resident cells, which in turn drives further remodeling.

**Figure 1:**
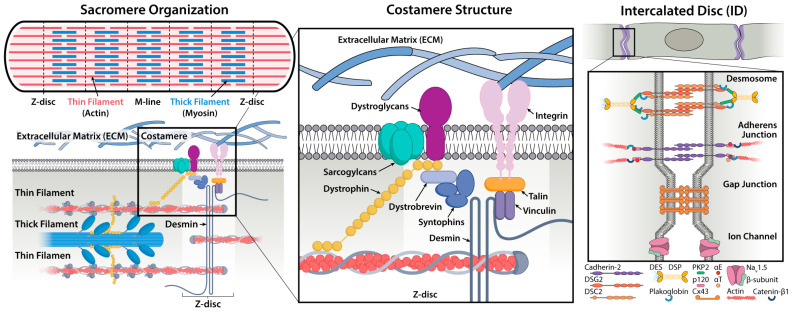
**Cardiomyocyte cell–cell and cell–ECM connections are coupled to contractile machinery.** Costameres connect cardiomyocytes to the ECM at the Z lines for force transmission (**left**). The mechanosensitive costamere includes focal adhesion proteins (integrins, talin, and vinculin) and connects to the thin filaments via desmin (**middle**). Intercalated discs connect neighboring cardiomyocytes through gap junctions, adherens junctions, and desmosomes. Adherens junctions connect adjoining cardiomyocyte cytoskeletons to enable force transmission (**right**).

In cardiomyocytes specifically, focal adhesion functions are performed by costameres, which are specialized adhesion structures that link sarcomeric Z-lines to the ECM (Figure 1) [48,49,50]. These bidirectional mechanotransductive structures transmit contractile forces outward while also relaying external mechanical cues back to the contractile machinery. Costameres dynamically remodel in response to substrate stiffness, cyclic stretch, and topography, meaning that changes in ECM mechanics are continuously registered and translated into adjustments in contractile output [48,51,52,53,54]. Disruption of this bidirectional coupling distorts mechanosensing, leading to dysregulated feedback and maladaptive remodeling, as observed in pathological states with impaired integrin-mediated adhesion [25,55,56,57].

### 2.3. Cell–Cell Junctions and Integrated Mechanical Networks

Mechanoreciprocity requires responses to ECM cues by individual cells and the spread of these signals to create coordinated tissue-level responses. Cardiomyocytes are connected through gap junctions, adherens junctions, and desmosomes, which are concentrated at intercalated discs, allowing for electrical propagation and force transmission between cells (Figure 1).

Adherens junctions physically link the actin cytoskeletons of neighboring cells though N-cadherin based complexes, and support the transmission of strong contractile forces between cells and across tissue, enabling force-dependent cytoskeletal remodeling to propagate beyond the originating cell [58,59]. Desmosomes, which connect intermediate filaments between cells, reinforce this structural coupling (Figure 1); however, disruption of these junctions weakens intercellular adhesion and contributes to pathological remodeling [60,61]. The electromechanical syncytium relies on coordinated ionic movement controlled by key gap junction proteins such as connexin43 (Cx43) that regulate intercellular electrical coupling (Figure 1) [62]. Loss or abnormal localization of gap junctions disrupts cardiomyocyte electrical coupling [63,64,65,66,67].

In the context of mechanoreciprocity, cell–cell junctions serve a critical propagating function allowing mechanical perturbation sensed by one cell, whether a change in ECM stiffness, fiber alignment, or stretch, to be communicated to neighboring cells through both structural linkages and ionic coupling. Meaning, local remodeling events can generate tissue-level mechanical consequences, and conversely, tissue-level mechanical changes can redistribute mechanical load across many cells simultaneously. The intercalated disc, therefore, acts as a signal integration and broadcast node within the multicellular feedback system.

## 3. Cardiomyocyte Interpretation of ECM Mechanics

Through integrin-based focal adhesions, cardiomyocytes continuously sense ECM stiffness and organization in terms of contractile activity and hemodynamic loading, adjusting their structural and functional state accordingly. Matrix stiffness is a biologically instructive cue. For example, compared to stiffer substrates (17.3 kPa), softer matrices (0.68 kPa) promote positive chronotropism, increased calcium homeostasis, as well as more cell maturation and adhesion protein expression [51,68]. In contrast, cardiomyocytes cultured on stiffer or disorganized substrates generate less contractile stress and exhibit impaired myofibril alignment [51,69]. Matrix organization promotes maturation, evidenced by increased expression of ventricular myosin light chain isoforms, reduced expression of fetal troponin T, and decreased phosphorylation of alpha tropomyosin in hiPSC-CMs on chevron micropatterned substrates [70]. These responses are controlled by multiple mechanotransductive pathways that must cooperate for proper cytoskeletal remodeling and cell growth [71].

Importantly, cardiomyocytes exhibit mechanical memory, where exposure to elevated stiffness (~50 kPa) results in cytoskeletal rearrangements that can remain even after a new physical cue has been added [72]. For instance, Vite found similarities between peak velocity rates and peak calcium in stiff and softening conditions [72]. In cardiomyocytes, this persistence is dependent on microtubule dynamics and is also suggested to rely on talin stability, a key regulator of cardiac cell adhesions [73,74,75,76]. It is hypothesized that these long-lived talin-dependent signals are linked to the epigenetic imprinting that is partially responsible for mechanical memory [74]. The additional work required to fully understand mechanical memory in cardiomyocytes, including the duration and specific proteins involved in this cytoskeletal rearrangement, will depend on advanced models that can integrate both cellular and extracellular contributions in a dynamic mechanical environment.

Beyond structural effects, ECM elasticity and tissue architecture also regulate cardiomyocyte metabolism [77]. Increased stiffness elevates ATP production and basal respiration, likely reflecting increased energetic demand required to overcome mechanical resistance, while matrix alignment enhances metabolic adaptability [77]. Substrate anisotropy also influences gene expression, including atrial natriuretic peptide expression in cultured cardiomyocytes [78]. Increased substrate stiffness (0.2 to 50 kPa) caused a loss of coherent electrical oscillation and ion channel dysregulation (cycle length from ~600 to 1300 ms) in avian embryonic sinoatrial node pacemaker cells [79]. Collectively, these stiffness-dependent changes impair contractility, maturation, calcium handling, and electrophysiologic coupling, directly connecting mechanosensing to reduced cardiac output and increased risk of developing arrhythmias. These cardiomyocyte-driven mechanical alterations provide cues that other cardiac cell types, including fibroblasts and immune cells, sense and respond to.

## 4. Fibroblasts as Amplifiers of Mechanical Feedback

### 4.1. Fibroblast Mechanosensing

As responders to cardiomyocyte-driven mechanical cues, cardiac fibroblasts are key mediators of ECM remodeling, sensing stiffness and stretch through integrins, mechanosensitive ion channels, and actin-dependent YAP/TAZ signaling. Cardiac fibroblasts, which are associated with ECM homeostasis, can be activated to a myofibroblast phenotype through autocrine, paracrine, hormonal, or mechanical signaling. Myofibroblasts express genes associated with inflammatory signaling, mechanotransduction, and ECM remodeling [80]. They dramatically remodel the ECM by depositing excessive structural proteins, including collagen, fibronectin, laminins, and others, as well as matrix-degrading enzymes known as matrix metalloproteinases (MMPs) [24,81]. Highly contractile myofibroblasts also secrete tissue inhibitors of MMPs (TIMPs), contributing to the complex control of matrix turnover.

Fibroblast activity can be altered by biochemical and mechanical cues in ways that can be cooperative or competitive [82,83]. Substrate stiffness modulates fibroblast activation, with stiff substrates (41 kPa) causing activation and soft substrates (6 kPa) inducing quiescence [37,84]. Aortic fibroblasts exhibit altered phenotype and sensitivities to mitogen-activated protein kinase inhibitors depending on substrate stiffness [85,86]. Alignment of collagen fibrils within the matrix also increases fibroblast activation in a contractile-dependent manner [87]. Integrins play a key role in fibroblast activation and persistence. For example, inhibition of ITGα5 and ITGβ5 improved cardiac function and decreased markers of fibroblast activation [88,89,90]. Specific integrin subunits can alter cardiac fibroblast activity; specifically, β1 integrins are critical for remodeling, and the β3 subunit promotes cardiac fibrosis [91,92]. Pressure (pneumatically applied as compressed air ~19 kPa) and stretch (5% strain) can activate fibroblasts, increasing collagen gene expression [93]. Stretch (6.5% strain), in combination with a panel of profibrotic agonists that act through distinct receptor systems (GPCRs, cytokine receptors, and receptor tyrosine kinases), can amplify, dampen, or reverse biochemical stimuli depending on the specific agonist treatment used [82]. One specific agonist, TGFβ-1, which binds multiple receptors in cardiac fibroblasts, cardiomyocytes, endothelial cells, and smooth muscle cells, is a key driver of fibroblast activation, which causes changes in migration, cell adhesion, and ECM remodeling; TGFβ-1 can be released by fibroblasts themselves or other cell types [88,94]. Secretion of TGFβ-1 by various cardiac cell types occurs in a stage-dependent manner, which results in an autocrine and paracrine amplification loop. This feedback loop modulates collagen synthesis, ECM remodeling, and fibroblast persistence, all important drivers of overall cardiac function. However, studying these multicellular TGFβ-1 interactions requires a system capable of cyclic changes in mechanical properties (on the frequency order of 1–5 Hz) and is difficult to understand outside of the mechanoreciprocity framework.

Cardiac fibroblasts also exhibit mechanical memory, partially through changes in gene expression and cytoskeletal organization that remain up to 96 h after the mechanical cues have changed [94,95]. Fibroblast activation state can be maintained when seeding them on either soft or stiff surfaces and transitioning them to the other [96]. Cardiac fibroblasts transfer their F-actin-dependent mechanical memory to the ECM through collagen alignment and tension [76,97].

### 4.2. Alignment and Long-Range Force Transmission

Myofibroblasts actively remodel collagen matrices by exerting contractile forces on the fibers through integrin-mediated adhesions [23]. Tension generated by these forces drives fiber alignment, enabling regional force transmission (250–1300 µm) [33,98]. Aligned fibers guide cell orientation, which further contributes to the alignment of deposited collagen, creating a positive feedback loop. In fibrillar collagen matrices, myofibroblast contraction creates deformation fields that influence the behavior of other cells, including macrophages at distances 20–40 times larger than their diameter, demonstrating that force transmission through the ECM is not localized to the immediate cellular environment [33,98]. Myofibroblasts alter collagen network compaction, alignment, and fibril bending, which are sensed by adherent cells as mechanical cues, including strain, stiffness gradients, and topographies [98]. Through collagen deposition, alignment, and crosslinking, fibroblasts contribute to either pro- or anti-fibrotic pathways both directly and indirectly by influencing cardiac macrophages’ behavior.

## 5. Macrophages as Regulators of Immune-Mechanical Feedback in Remodeling

### 5.1. Homeostatic Roles

CCR2^neg^ cardiac resident macrophages respond to fibroblast-driven ECM mechanical cues, clear debris, and maintain tissue homeostasis. Macrophages remove subcellular vesicles, called exophers, that are released from cardiomyocytes, which contain dysfunctional mitochondrial cargo [99]. Efficient ingestion of this cellular waste is crucial because it prevents accumulation, inflammasome activation, and autophagic block [99]. They also work to facilitate electrical conduction of the AV node through gap junctions with cardiomyocytes [99,100,101].

Macrophages within the heart interact with cardiomyocytes through mechanosensitive focal adhesions [102]. Specifically, CCR2^neg^ macrophages can be activated through the membrane stretch-activated transient receptor potential vanilloid 4 (TRPV4) channel and express pro-angiogenic growth factors [102]. The TRPV4 channel is opened due to macrophage membrane stretching (1 Hz, 10% deformation for 24–48 h), which can be caused by cardiomyocyte contraction [102]. Recent in vivo work further demonstrates coordinated interactions between cardiomyocytes, macrophages, and the ECM during cardiac regeneration in some model systems, where macrophage-dependent ECM remodeling enables cardiomyocyte invasion into injured tissue [22]. Integrin expression can be controlled by signaling cascades; specifically, CD74-MIF can trigger pathways that, when inhibited, promote tissue damage and fibrosis [103].

In addition to cell-derived mechanical stretch, macrophage phenotype can be directly influenced by matrix stiffness sensed through cell–ECM adhesions [104,105,106,107]. Specifically, macrophage pro-inflammatory marker expression increases with increases in stiffness from 0.2 to 280 kPa [105,106,107]. Similar to other cell types, macrophages show increased spread, F-actin assembly, and localized integrin expression on stiff compared to soft substrates (compressive moduli of 840 vs. 130 kPa) [108]. Aligned fibers (15 to 45°) can reduce pro-inflammatory marker expression, eliciting a more anti-inflammatory response [109,110]. Furthermore, matrix fiber diameter can alter macrophage phenotype, with larger fibers causing increased pro-inflammatory marker secretion compared to smaller ones (~1.6 vs. ~0.58 µm) [111]. Macrophages are mechanosensitive to stretch, pressure, and ECM stiffness, organization, and composition, which regulate their inflammatory phenotype.

### 5.2. Immune–Fibroblast Crosstalk

In addition to crosstalk with cardiomyocytes, macrophages interact with fibroblasts to regulate ECM remodeling through both secreted factors and direct cell contact. Frangogiannis documented that macrophages and mast cells contribute to the proteolytic environment in pressure-overloaded and ischemic hearts through MMP secretion, promoting fibroblast activation and ECM expansion as downstream consequences of injury [20]. In the mechanoreciprocity framework, however, these immune–ECM interactions are not simply injury responses but constitute alterations to an ongoing feedback loop in which mechanical cues continuously modulate macrophage phenotype, which in turn regulates fibroblast activity and matrix remodeling. Macrophages and fibroblasts interact through secreted factors that control the balance of fibrillogenesis and fibrolysis and shape matrix formation and degradation [112]. Pro-inflammatory like macrophages, can cause myofibroblast transition. Increased luminal pressure (afterload) causes a subpopulation of macrophages to express CCL24, which activates fibroblasts through CCR3, causing TGF-β release and a further cascade of fibroblast activation [112]. Macrophage-secreted TNF-α and IL-1β could also contribute to fibroblast activation seen in injury states, including ischemic cardiomyopathies and myocardial infarction [113,114]. Inflammatory responses associated with macrophages correlate with upregulation of remodeling molecules (MMP2, MMP9, and collagen) and a downregulation of homeostasis markers (TIMP2) [20,113]. Macrophages and fibroblasts can also directly interact through integrins; for example, profibrotic macrophages can trigger acute fibroblast contraction that is dependent on αvβ3 in macrophages and Piezo1 in fibroblasts [115]. This macrophage-dependent fibroblast activation can even override mechanical cues, lasting even in soft environments [115]. Others have also demonstrated that biochemical signals can override mechanical ones, showing M2 macrophages can activate fibroblasts on soft hydrogels through IL-6-dependent signaling [116]. In addition to matrix remodeling, myofibroblasts can aid macrophages with phagocytosis by secreting milk fat globule-epidermal growth factor (MFG-E8) after myocardial infarction [19,117]. Macrophages and fibroblasts can cooperate to increase either pro- or anti-inflammatory responses, but a full understanding of these cell interactions and responses requires studying them within the overall biomechanical regulatory system. The resulting changes in cardiac tissue mechanical properties, a complex set of interactions involving multiple cell types, may be best studied through the lens of mechanoreciprocity.

### 5.3. Contributions to Maturation in Engineered Systems

The incorporation of macrophages into engineered heart tissues improves the overall functionality of these systems through multicellular interactions with cardiomyocytes and fibroblasts. For example, macrophages can improve contractile strength, relaxation kinetics, maturation, long-term vascularization, and reduce cytotoxicity [100,118,119,120]. Macrophages can upregulate expression of cardiomyocyte sarcomeric and desmosomal proteins, matrix remodeling markers, and pro-angiogenic genes [118,119]. However, direct cell–cell contact is not required, as embryonic-derived cardiac resident macrophage conditioned media can stimulate cardiomyocyte proliferation [30]. Macrophages, therefore, serve as mechanical immunomodulators, capable of amplifying or dampening fibroblast-driven remodeling in response to altered tissue strain.

## 6. Additional Cell Types Involved in Cardiac Mechanoreciprocity

Beyond cardiomyocytes, fibroblasts, and macrophages, other cell types, including endothelial cells, pericytes, and vascular smooth muscles, also play key roles in sensing and translating mechanical cues. While a comprehensive discussion is outside the scope of this review, we provide a brief consideration highlighting their roles in integrating hemodynamics and matrix-derived signals. Endothelial cells respond to shear stress and substrate stiffness, with changes in flow (4–12 dyn/cm^2^) altering adhesion and angiogenic gene expression, including VEGF-A and TGF-β1 [121,122,123]. Pericytes, which are mechanically coupled to endothelial cells and the basement membrane, experience circumferential stretch and shear stresses from fluid flow [124]. Pericytes increase inflammatory cytokines and ECM remodeling proteins in stiff environments (~28 kPa), promoting vascular inflammation and remodeling, further progressing fibrotic cascades [125]. Similarly, vascular smooth muscle cells respond to ECM stiffness (2–25 kPa) by modulating phenotype, contractility, and migration [126]. These vascular-associated cells integrate hemodynamic forces with ECM remodeling and inflammatory signaling, extending mechanoreciprocity beyond the myocardium. Rather than acting as independent drivers, they establish boundary conditions that regulate cardiac cell mechanical inputs, reinforcing feedback between vascular and myocardial cell types. Dysregulation of this mechanoreciprocity can contribute to pathological remodeling.

## 7. Multicellular Mechanoreciprocity: Interdependent Feedback Loops

**Figure 2:**
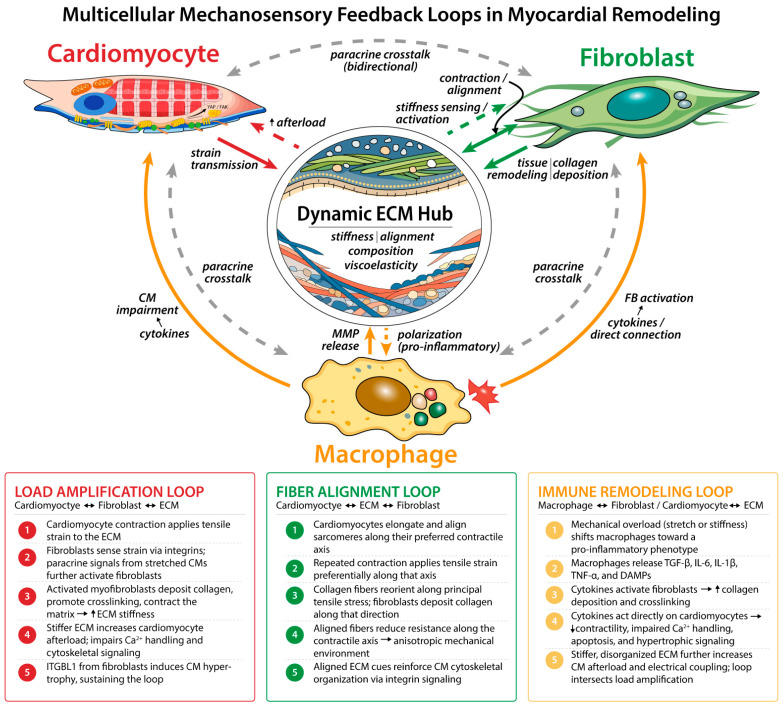
**Multicellular Mechanosensory feedback loops in myocardial remodeling**. The load amplification loop (red) is driven by cardiomyocyte-induced ECM strain that activates fibroblasts, which stiffen the matrix and increase afterload, a positive feedback cycle impaired by elevated ECM stiffness. The fiber alignment loop (green) emerges from preferential force transmission along the cardiomyocyte contractile axis, directing anisotropic collagen deposition that reinforces cellular alignment. The immune remodeling loop (orange) is initiated by mechanosensitive macrophage polarization; pro-inflammatory cytokines (TNF-α, IL-1β, IL-6, TGF-β) act both indirectly, by activating fibroblasts and stiffening the ECM, and directly on cardiomyocytes, impairing contractility, Ca^2+^ handling, and promoting apoptosis. Solid arrows indicate direct activating relationships; dashed arrows indicate mechanosensory or paracrine feedback. CM, cardiomyocyte; ECM, extracellular matrix; ITGBL1, integrin beta-like 1; DAMP, damage-associated molecular pattern. Double sided arrow indicates reciprocal relationship between cells, right facing arrow represents causation from the first action to the second, and upward arrow denotes an increase.

Prior reviews have addressed many of the individual components underlying cardiac ECM remodeling. Frangogiannis provided a comprehensive account of ECM macromolecule dynamics in ischemic and nonischemic heart failure, discussing how fibroblasts, immune cells, and cardiomyocytes contribute to matrix remodeling across distinct injury states [20]. However, these interactions are organized by injury type and ECM composition rather than as interdependent feedback loops, and mechanosensing is not framed as a continuous reciprocal process operating across cell types under both homeostatic and pathologic conditions. More recent in vivo work has investigated how these interactions coordinate within intact tissue. Specifically, macrophage-driven ECM remodeling, mediated in part through mmp14b expression in a border-zone macrophage subpopulation, is required for cardiomyocyte protrusion into injured tissue during cardiac regeneration in zebrafish, providing direct evidence of coordinated cardiomyocyte–macrophage–ECM interactions in vivo [22]. However, these studies address specific mechanisms in defined injury or regenerative contexts rather than organizing multicellular mechanical interactions into a systems-level framework. Here, we reorganize these interactions into three interdependent feedback loops, load amplification, structural alignment, and immune remodeling, which collectively control whether the myocardium maintains homeostasis or progresses toward maladaptive remodeling (Figure 2).

### 7.1. Load Ampification Loop

The load amplification loop describes a feedback system where cardiomyocyte-generated forces deform the ECM and are sensed by fibroblasts, which respond by remodeling the matrix, increasing mechanical load, and therefore altering cardiomyocyte function. Prior models have treated cardiomyocyte force generation and fibroblast-driven matrix remodeling as largely separate processes [20,127,128], whereas the load amplification framework explicitly links these interactions as a coupled feedback system.

The load amplification loop involves the interactions between cardiomyocytes, fibroblasts, and the ECM. As cardiomyocytes contract, they pull on the ECM, straining the matrix. Cardiac fibroblasts then sense this strain through integrins, YAP, and mechanosensitive ion channels such as Piezo1 and promote profibrotic signaling. For example, adeno-associated viral delivery of YAP to neonatal mice increases mRNA expression of Col1a1, Col3a1, TGFβ-1, and fibronectin, and increases the number of α-SMA positive cells [129]. Piezo1 activation in human cardiac fibroblasts increases IL-6 production, another profibrotic marker [130]. The role of mechanosensors in stiffness-induced fibrotic behavior is further supported by the reduction in fibrotic markers following YAP inhibition [131]. Manipulation of mechanosensors in fibroblasts supports the concept that cardiomyocyte-derived strain on the ECM activates fibroblasts.

Paracrine signaling from stretched cardiomyocytes, direct stretch of the fibroblasts, or matrix stiffening can activate fibroblasts [132]. Through remodeling, myofibroblasts increase the amount of force required to deform the ECM and the afterload that cardiomyocytes must work against (Figure 2) [81]. This increase in stiffness impairs cardiomyocyte contractility and calcium handling by modifying cytoskeletal and mechano-dependent signaling [72]. Specifically, rat adult cardiomyocytes on stiff substrates (54.3 kPa) showed reduced contractile velocity, peak [Ca^2+^]_i_, prolonged calcium reuptake, and increased cardiomyocyte viscoelasticity and microtubule detyrosination [72]. Cardiac fibroblasts can also induce hypertrophy in cardiomyocytes and promote fibroblast activation through the release of integrin beta-like1 and IL-6 [130,133].

Importantly, not all cardiomyocyte–fibroblast crosstalk impairs cardiac function. Cardiac fibroblasts can improve calcium handling, increase contractile strains, promote cardiomyocyte maturation, and improve mitochondrial respiration maturity [134,135,136,137]. Overall incorporation of fibroblasts into in vitro studies has improved structural, electrical, mechanical, and metabolic maturation of cardiomyocytes [137]. However, age can alter this crosstalk; adult cardiac fibroblasts decreased electrophysiological and mechanical function compared to fetal fibroblasts, which improved or did not affect these functions [138].

### 7.2. Structural Alignment Loop

The structural alignment loop describes a feedback system in which force generation and transmission drive anisotropic ECM organization, which in turn reinforces directional cellular alignment and force propagation.

Load-induced ECM alterations thereby trigger the alignment loop, which involves coordinated organization of cardiomyocytes and collagen fibers within the ECM. Cardiomyocytes elongate along a preferred axis, which corresponds with sarcomere alignment and the direction of maximal contractile forces [139]. This repeated contraction generates cyclic tensile strain along that axis, establishing a principal direction of stress. This concept has been documented in many studies, for example, human embryonic stem cell-derived cardiomyocytes align in the direction transverse to uniaxial cyclic stretch (10–30% elongation in length at 1 Hz) in a TRPV4-dependent manner [140]. However, both cardiomyocytes and fibroblasts reorient their cytoskeletons along the direction of principal tensile stress. Fibroblasts preferentially deposit and remodel collagen along these aligned fibers, reinforcing ECM anisotropy.

Contraction, therefore, creates a more structurally aligned matrix, and mechanical resistance becomes direction dependent. This enables more effective transmission of cardiomyocyte force along aligned fibers. Together, cyclic contraction drives ECM alignment, providing anisotropic mechanical cues that reinforce cardiomyocyte alignment, creating a positive feedback loop that enhances ECM and cell alignment.

### 7.3. Immune Remodeling Loop

The immune remodeling loop describes how mechanosensitive macrophages respond to changes in ECM mechanics to regulate fibroblast activation and cardiomyocyte function through inflammatory signaling, forming a feedback system that links mechanical and immune responses.

In addition to cardiomyocytes and fibroblasts, macrophages are also altered by these mechanical cues, triggering the immune remodeling loop where signals are spread to other cell types. Mechanically activated pro-inflammatory-like macrophages release cytokines and damage-associated molecular patterns (DAMPs), including TGF-β, IL-6, IL-1β, and TNF-α, activating fibroblasts and indirectly increasing collagen deposition and crosslinking (Figure 2) [105,106,141]. This macrophage-derived fibroblast activation can depend on direct interactions. Specifically, a fibroblast-specific deletion of CCR3 that binds to cardiac resident macrophages reduced fibrosis after transverse aortic constriction-induced pressure overload in mice [112].

Macrophage-induced myofibroblast differentiation reinforces ECM stiffening, directly relating to the load amplification loop. Cardiac resident macrophages can interact with cardiomyocytes through cytokines, including IL-1β and TNF, which have been shown to alter Cx43 location in iPSC-derived cardiomyocytes, impairing electrical conductivity within the myocardium [142]. Thus, mechanical overload reshapes macrophage behavior, which in turn alters macrophage signaling and fibroblast activation, cardiomyocyte hypertrophy, and tissue electrophysiology [20]. This loop reinforces the idea that the immune system is not only a part of homeostasis but also plays a role in mechanical stress and structural remodeling within the heart.

### 7.4. Integration of Interdependent Loops

These multicellular feedback loops are interdependent regulators of myocardial homeostasis. Cardiomyocytes, fibroblasts, and macrophages exchange both biochemical and mechanical signals that propagate throughout the tissue, starting with microscale matrix remodeling and ultimately leading to organ-level dysfunction. When the feedback loops are balanced, they support structural and functional homeostasis; in contrast, disruption drives fibrosis, electrical instability, and contractile dysfunction. Over time, maladaptive feedback contributes to the progression of cardiomyopathies and eventual heart failure without intervention. Importantly, the three loops do not operate independently but are dynamically coupled, such that changes in one loop propagate across the system to influence the others, forming an integrated multicellular feedback network.

## 8. Engineered Cardiac Systems as Platforms to Study Mechanoreciprocity

Studying these loops requires engineered systems that recapitulate key mechanical and structural features of the myocardium. Engineered models of myocardial tissue using human cells provide diverse and increasing opportunities for basic biological research, preclinical pharmacology [143], and disease modeling [144]. In theory, the ability to control and vary key inputs should empower identification of mechanisms regulating tissue function in a manner that is superior to in vivo experimentation. Likewise, appropriate multicellular composition, architecture, and relevant biomechanical inputs should yield in vivo relevance that exceeds that of conventional cell culture models.

Accurate modeling of the native myocardium requires a highly organized, anisotropic structure primarily made of collagen. Matrix elastic moduli must also fall within physiologically meaningful ranges for healthy or fibrotic pathological states. Materials used to model native myocardium should incorporate time-dependent (viscous) mechanical behavior rather than purely elastic responses. Platforms designed to model disease progression further benefit from tunable or dynamically adjustable stiffness to mimic matrix remodeling over time.

Myocardium comprises at least nine major cell types [145]. Cardiomyocytes are relatively large contractile cells and comprise 70–85% of myocardial volume and 25–35% of all cells in the adult heart [103]. Recent evidence indicates that 15–25% are fibroblasts and 3–8% are immune cells [103]. Engineered heart models with multiple cell types, including immune cells, have better functional outcomes, provide information about cellular crosstalk, and have improved relevance.

### 8.1. Two-Dimensional Models

Advances in material engineering have enabled substrates with tunable and dynamically adjustable stiffness, allowing mechanical cues to be varied independently of biochemical signals. Dynamic stiffness modulation has been achieved using secondary chemical crosslinking [146,147,148,149,150], thermoresponsive polymers [151,152], and light-activated (photodegradable or photocrosslinkable) hydrogels [153,154,155,156]. These approaches have enabled temporal control over matrix mechanics and have been instrumental in studying adhesion remodeling [157,158], cytoskeletal adaptation [158,159], and mechanical memory [72,76]. However, many of these systems produce relatively slow, unidirectional, and often irreversible transitions (e.g., soft→stiff), and light-patterned platforms have largely been limited to softer regimes (<~6 kPa) and modest stiffness amplitudes [72]. Such constraints limit their ability to model the full mechanical spectrum of adult and fibrotic myocardium.

Magnetorheological elastomer (MRE) platforms provide an alternative strategy. By embedding magnetic particles within a polymer matrix, stiffness can be rapidly, reversibly, and repeatedly modulated through application or repositioning of an external magnetic field [72,76,83,158,160,161]. Because the underlying network structure remains intact, MRE-based systems enable real-time, bidirectional stiffness control across physiologically relevant ranges, making them particularly well suited for studying dynamic mechanical environments and feedback processes central to mechanoreciprocity.

Cyclic or static strain can also be incorporated to mimic preload, pressure overload, or volume overload [82,162,163,164]. These platforms typically use flexible membranes actuated pneumatically or mechanically, allowing modulation of strain amplitude, frequency, and waveform. Cyclic stretch has been instrumental in studying sarcomere organization [165,166], focal adhesion rearrangement [167,168], cytoskeletal alignment [169,170], and stretch-activated signaling pathways [82,162,171] in both cardiomyocytes and fibroblasts. Importantly, strain can be applied uniaxially or biaxially, enabling interrogation of anisotropic force transmission. However, in most 2D stretch systems, substrate stretch and substrate stiffness are often experimentally coupled, complicating isolation of purely strain-dependent effects.

Micropatterning and surface topography provide spatial control over cell geometry and alignment. Dynamic alterations using micro/nanogrooves [158,172,173,174,175], fiber alignment [176,177], and surface wrinkling [40,178,179] can direct cytoskeletal organization, focal adhesion distribution, and anisotropic force generation. These approaches have been particularly valuable for imposing cardiomyocyte alignment, improving sarcomere organization, and standardizing cell shape to reduce variability in mechanotransduction studies. Emerging strategies allow dynamic modification of surface architecture, enabling investigation of how cells respond to evolving geometric cues. For instance, strain-induced wrinkling systems typically consist of a stiff thin film layered atop a compliant elastomer. When stretched and released, mechanical instability generates reversible wrinkle patterns whose wavelength and amplitude can be tuned by strain magnitude and material properties. Shape-memory polymers provide another approach, in which surfaces are temporarily programmed into one topographic state (e.g., flat) and later triggered (e.g., heat-activated) to recover a pre-defined patterned configuration, dynamically altering ridge depth or spacing beneath adherent cells. Light-responsive materials offer spatiotemporal precision by incorporating photolabile or photocrosslinkable chemistries that locally alter crosslink density or induce surface buckling upon illumination, enabling patterned or reversible changes in microfeatures without mechanical actuation. Finally, MREs allow magnetic field-induced modulation of surface microfeatures, whereby applied fields increase the prominence of existing topography in a reversible and controllable fashion [158]. 2D platforms allow for interrogating how cardiac cells read, integrate, and rewrite mechanical inputs prior to translation into 3D models.

### 8.2. Three-Dimensional Engineered Tissues

Compared to 2D models, 3D engineered tissues more faithfully recapitulate the multicellular architecture, matrix confinement, and force transmission present in native myocardium. By embedding cells within a 3D matrix, these systems enable volumetric force generation, cell–cell coupling, and matrix remodeling in all spatial dimensions, capturing emergent behaviors not observed in planar culture. 3D cardiac models more commonly emphasize dynamic mechanical loading, whereas 2D systems primarily manipulate substrate stiffness. In the native heart, mechanical regulation occurs beat-to-beat through cyclic stretch, preload, afterload, and pacing frequency; engineered tissues, therefore, incorporate controllable boundary conditions to replicate these physiological and pathological cues.

Mechanical load in 3D constructs acts as an active regulator of tissue maturation and remodeling. Increasing afterload promotes cardiomyocyte maturation, including enhanced sarcomere organization, cell elongation, and improved calcium handling [180]. However, sustained elevation beyond physiological thresholds induces pathological hypertrophy and fibrotic remodeling [180]. Similarly, cyclic stretch used to model preload, particularly in multicellular constructs containing cardiac fibroblasts, enhances maturation and improves contraction kinetics [163]. These findings underscore that mechanical inputs in 3D systems do not merely constrain contraction but drive structural and functional adaptation.

Different 3D platforms regulate preload and afterload through distinct mechanical architectures. Cantilever-based engineered heart tissues (EHTs) suspend microtissues between flexible posts that serve as anchors [180,181,182,183,184,185,186,187,188,189]. Tissue compaction establishes preload, while post stiffness defines afterload and enables quantitative force measurement via post deflection. Biowire systems, in contrast, rely on a central suture that promotes cardiomyocyte alignment and anisotropy; preload develops through compaction, but afterload is less precisely tunable [190,191]. Hybrid configurations such as Biowire II combine alignment cues with defined mechanical anchoring to improve load control [190,191]. Microfluidic platforms embed tissues within perfused channels, supporting metabolic supply while incorporating microposts or stretch mechanisms to regulate load [192,193]. Sacrificial mold-based multistrip constructs generate aligned tissue strips within defined geometries, where preload arises from compaction and afterload modulation typically requires external actuation [194].

Collectively, these 3D engineered tissue platforms interrogate how preload, afterload, and pacing frequency coordinate multicellular remodeling and mechanoreciprocal feedback at the tissue scale. Fine-tuning these parameters enables researchers to model both physiologic and pathologic conditions, isolating specific mechanisms regulated by dynamic mechanical environments, informing the development of targeted therapeutic interventions.

**Figure 3:**
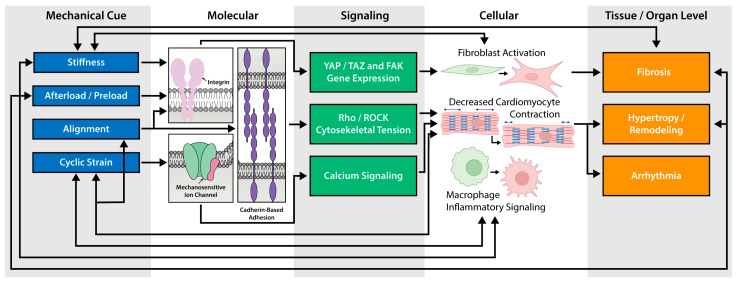
**Multiscale integration of mechanical cues**. Mechanical cues encoded by the ECM are sensed through integrins, mechanosensitive ion channels, and cadherin-based adhesions. Specific cues and molecular structures activate unique signaling pathways that alter cellular behavior. Interactions between fibroblasts, cardiomyocytes, macrophages, and other cell types caused by signaling changes can lead to tissue/organ-level dysfunction. Various cell functions and pathological conditions can also impair mechanical cues, represented by double-sided arrows, highlighting the central theme of mechanoreciprocity.

Precise modulation of specific mechanical cues allows engineered platforms to model distinct pathological states to study cell behavior and mechanisms. For example, increased elastic modulus as observed in fibrotic remodeling, post-myocardial infarction scars (35–70 kPa), and hypertrophic cardiomyopathy (G ~12.7 kPa) (Figure 3) can be easily modeled with current platforms [195,196,197]. Topographical cues, including cell alignment, modulate cell geometry, action potential conduction, and cell–cell coupling proteins, with disruption of alignment reducing force transmission [198,199]. Altered alignment is a key characteristic of hypertrophic cardiomyopathy and the post-myocardial infarction scar (degree of alignment from 0.04–0.44) (Table 1) [200,201]. Cyclic stretch regulates cytoskeletal arrangement and calcium dynamics, promoting either adaptive or maladaptive remodeling based on magnitude and duration (Figure 3) [165,202,203]. Pressure overload, seen in hypertrophy and aortic stenosis, increases afterload, resulting in hypertrophic remodeling and matrix stiffening (Figure 3) [20,204,205]. Volume overload, characteristic of mitral regurgitation, results in ventricular dilation and impaired strain (Table 1) [20,206]. Together, these mechanical inputs control contractile function, electrophysiology, and progression towards heart failure that can be recapitulated using in vitro models.

Despite multiple advances in the field, no single current engineered system can fully recapitulate in vivo mechanoreciprocity. Instead, existing systems offer complementary strengths. 2D systems offer precise control over substrate stiffness, ligand presentation, and cell geometry, enabling reductionist interrogation of adhesion signaling and mechanostransduction. However, these 2D models lack native complexity, making it challenging to include multiple mechanical cues within the same system or multicellular interactions. 3D systems better mimic tissue-level mechanics, including force generation, preload, afterload, and multicellular remodeling dynamics, but are limited in throughput, precision, and temporal control. Many models do not fully incorporate immune components, spatial heterogeneity, or long-term ECM remodeling. Isolating individual parameters remains challenging, as altering one mechanical cue often unintentionally affects another. Additionally, variability across studies in terms of cell types, matrix composition, mechanical readouts, and lack of standardized metrics makes it difficult to directly compare results. Further development is required to study long-term or complex mechanoreciprocal interactions.

Future studies must better recapitulate the dynamic multidimensional nature of the cardiac mechanical environment. Many studies investigate isolated mechanical cues such as stiffness; however, cells integrate multiple parameters simultaneously, including viscoelasticity, matrix alignment, composition, and cyclic strain. With this, there is a need for platforms that can independently tune and combine these cues with physiologically relevant values. Progress in this area depends on improved ECM characterization in both healthy and pathological states. Additionally, current culture systems remain limited in their ability to support multicellular interactions required to capture cardiomyocyte, fibroblast, and macrophage crosstalk. Engineered systems that incorporate multiple cell types and allow cell-mediated matrix remodeling will be critical for modeling mechanoreciprocal signaling. Rather than isolating specific mechanostransduction pathways, future work should aim to understand connected signaling networks and the subsequent cellular responses to complex mechanical inputs. Combining these platforms with omics approaches will further enable the identification of mechanosensitive signaling networks and cellular phenotypes. Integrating these approaches from molecular to tissue-level scales will be essential to understanding mechanisms of cardiac mechanoreciprocity and therapeutic applications.

## 9. Conclusions

The cardiac ECM is continuously remodeled and interpreted by cells, especially cardiomyocytes, fibroblasts, and macrophages, through coordinated mechanosensing and force transmission. These interactions establish multicellular feedback loops that govern tissue stiffness, alignment, and remodeling. Under physiologic conditions, these loops maintain mechanical homeostasis and are critical for adaptive functional maturation. In pathologic settings, the disruption or amplification of these feedback circuits contributes to progressive remodeling and fibrosis.

Framing cardiac remodeling as an interdependent mechanoreciprocal system shifts the focus of research from the study of isolated molecular pathways to a broader consideration of coordinated mechanical communication across cell types. Within this framework, cardiomyocytes generate mechanical inputs, fibroblasts amplify and restructure the matrix, and macrophages modulate inflammatory and remodeling responses. While these interactions have been studied individually, organizing them into coupled feedback loops provides a structure for understanding how local cellular behaviors scale to tissue-level dysfunction.

The multiple feedback loop perspective highlights several key unresolved questions. It remains unclear whether the load amplification, alignment, and immune remodeling loops operate with distinct temporal hierarchies or whether their relative contributions shift dynamically during disease progression. The existence of threshold conditions that drive transitions from adaptive to maladaptive feedback is not well defined, particularly in the context of stiffness- and strain-dependent fibroblast activation. In addition, it is unknown whether these loops can be experimentally decoupled or whether they are inherently interdependent under physiologic conditions. Finally, the extent to which mechanical memory is encoded and transferred across cardiomyocytes, fibroblasts, and macrophages within and between these loops represents a critical gap in understanding cardiac adaptation and disease. It also remains unknown whether loop thresholds, temporal dynamics, or the relative dominance of individual loops differ as a function of biological sex or age, both of which are associated with distinct baseline ECM compositions, inflammatory profiles, and susceptibilities to pathological remodeling.

Addressing these questions will require experimental systems capable of isolating and recombining key elements of mechanoreciprocity. In particular, dynamic material platforms that enable independent and reversible control of mechanical properties, including stiffness, viscoelasticity, and matrix organization, will be essential for interrogating how changes in mechanical inputs regulate feedback across cell types. Systems that decouple ECM alignment from bulk stiffness will be particularly important for dissecting the alignment loop and its role in anisotropic force transmission. Multicellular co-culture models incorporating cardiomyocytes, fibroblasts, and macrophages, with independent control over mechanical and inflammatory inputs, will allow investigation of immune–mechanical coupling and the extent to which biochemical signals can override mechanical cues. Time-resolved perturbation studies may further reveal how feedback loops are initiated, sustained, or reversed, while engineered tissues with closed-loop control of mechanical loading will be critical for modeling true mechanoreciprocal behavior.

Resolving these critical mechanistic questions has direct implications for therapeutic development. Rather than targeting individual molecular pathways in isolation, effective interventions may need to disrupt or reset the maladaptive feedback cycles that reinforce fibrosis, inflammation, and mechanical dysfunction. Such insight would drive therapeutic strategies to be both stage-specific and combinatorial, targeting dominant feedback processes at different phases of disease progression. In addition, measurable features such as ECM stiffness, alignment, and inflammatory state may serve as integrated biomarkers of loop activity, improving the prediction of disease progression and treatment response. Ultimately, integrating mechanobiological principles into therapeutic design may enable approaches that restore coordinated mechanical communication within the heart, rather than simply suppressing downstream pathological outcomes.

## Figures and Tables

**Table 1:** Key mechanical cues (e.g., stiffness, ECM alignment, stretch, and loading conditions) shape multicellular mechanoreciprocity in the heart by modulating cardiomyocyte, fibroblast, and macrophage behavior through changes in cellular signaling, structure, and function, ultimately driving disease-specific remodeling outcomes. Upward arrows indicate an increase and downward arrows denote a decrease.

Mechanical Cue	Cell Type(s)	Observed Cellular Response	Associated Pathology
↑ Stiffness	Fibroblasts, Macrophages, Cardiomyocytes	Fibroblast activation, inflammatory signaling, ↓ contractility, integrin clustering	Fibrosis, HFpEF
↓ ECM alignment	Cardiomyocytes and Fibroblasts	Diminished anisotropy	Arrythmia risk, impaired remodeling
Cyclic Stretch	Cardiomyocytes and Fibroblasts	Altered cytoskeleton and calcium handling	Adaptive vs. maladaptive hypertrophy
Pressure Overload	Cardiomyocytes	Concentric hypertrophy	Aortic stenosis, hypertension
Volume Overload	Cardiomyocytes	Eccentric hypertrophy, dilation	Mitral regurgitation

## Data Availability

No new data were created or analyzed in this study.
